# Pure platelet-rich plasma promotes semaphorin-3A expression: a novel insight to ameliorate intervertebral disk degeneration in vitro

**DOI:** 10.1186/s13018-023-04290-7

**Published:** 2023-10-20

**Authors:** Jie Huang, Shi-lin Lian, Jia-heng Han, Zheng-cao Lu, Yu Ding

**Affiliations:** 1 Orthopedics of TCM Senior Department, The Sixth Medical Center of PLA General Hospital, Beijing, 100048 China; 2 Department of Orthopedics, School of Medicine, South China University of Technology, Guangzhou, 510006 China; 3 Department of Orthopedics, School of Medicine, Jinzhou Medical University, Jinzhou, 121001 China

**Keywords:** Platelet-rich plasma, Leukocyte platelet-rich plasma, Pure platelet-rich plasma, Intervertebral disk degeneration, Sema3A, NF-κB signaling pathway

## Abstract

**Introduction:**

Intervertebral disk degeneration (IVDD) can be effectively treated using platelet-rich plasma (PRP). While the exact process is fully understood, it is believed that using pure PRP (P-PRP) without leukocytes is a better option for preventing IVDD. Semaphorin-3A (Sema3A), an inhibitor of angiogenesis and innervation, is essential for preserving IVDD’s homeostasis. Whether PRP prevents IVDD by modifying Sema3A has yet to receive much research. This work aims to clarify how P-PRP affects Sema3A when IVDD develops in vitro.

**Methods:**

Nucleus pulposus cells (NPCs) isolated from 8-week-old male Sprague-Dawley rats were exposed to 10 ng/ml IL-1β and then treated with P-PRP or leukocyte platelet-rich plasma (L-PRP) in vitro, followed by measuring cell proliferation, apoptosis and microstructures, inflammatory gene and Sema3A expression, as well as anabolic and catabolic protein expression by immunostaining, quantitative real-time polymerase chain reaction (qPCR), western blot, and enzyme-linked immunosorbent assay (ELISA).

**Results:**

In comparison with L-PRP, P-PRP had a higher concentration of growth factors but a lower concentration of inflammatory substances. P-PRP increased the proliferation of NPCs, while IL-1 relieved the amount of apoptosis due to its intervention. Anabolic genes, aggrecan, and collagen II had higher expression levels. MMP-3 and ADAMTS-4, two catabolic or inflammatory genes, showed lower expression levels. Sema3A activity was enhanced after P-PRP injection, whereas CD31 and NF200 expression levels were suppressed.

**Conclusions:**

P-PRP enhanced the performance of NPCs in IVDD by modifying the NF-κB signaling pathway and encouraging Sema3A expression, which may offer new therapy options for IVDD.

**The translational potential of this article:**

The findings provide a new therapeutic target for the treatment of IVDD and show a novel light on the probable mechanism of PRP and the function of Sema3A in the progression of IVDD.

## Introduction

Intervertebral disk degeneration (IVDD) is widely recognized as a major contributor to the prevalence of low back pain (LBP), which is extremely common throughout life and has a significant negative impact on hygienic and socioeconomic conditions worldwide [1–3]. As a multifactorial pathological process, IVDD is primarily characterized by an imbalance of extracellular matrix (ECM) anabolism and catabolism, which causes proteoglycan to be degraded and the water content of the nucleus pulposus (NP) to be consumed; other factors contributing to these diseases include inflammation and angiogenesis [4–6]. The intervertebral disk (IVD) consists of three distinct components: the inner nucleus pulposus (NP), the outer annulus fibrosus (AF), and the upper and lower cartilaginous endplates. These structures are vital for maintaining vertebral stability and protecting against mechanical stress [7, 8]. However, the avascular nature of the inner AF and NP tissues hampers their self-repair mechanisms, contributing to the degeneration of IVD tissues. Currently, conservative treatments for degenerative disk diseases (DDD), such as nonsteroidal anti-inflammatory medications, acupuncture, and physiotherapy, focus on providing symptomatic relief. Open and minimally invasive spine surgeries are frequently utilized to treat severe neurological problems brought on by herniated disks compressing nerve roots, and as a result, these procedures are thought to be a viable option for treating DDD [9]. Although removing the herniated disk and even spinal fusion, which further accelerates the advancement of IVDD treatment, may cause surrounding segmental disease and vertebral instability, this may be due to the neighboring disks degrading in a compensatory manner [10]. Therefore, the current therapy approaches cannot significantly slow the degenerative process.

Platelet-rich plasma (PRP) is a novel biological therapy that has garnered significant attention in both clinical and fundamental scientific research disciplines [11]. PRP is widely used in various orthopedic diseases, including bone, cartilage, ligament, tendon, and muscle, because of its better abilities for regeneration and restoration as well as high platelet concentrations above physiological levels [12, 13]. PRP, derived from autologous plasma, relies on functional growth factors such as platelet-derived growth factor (PDGF), transforming growth factor-beta (TGF-β), vascular endothelial growth factor (VEGF), insulin-like growth factor-1 (IGF-1), and basic fibroblast growth factor (bFGF) to mitigate or even reverse the progression of IVDD [14]. By biologically promoting cellular development, proliferation, and differentiation, the aforementioned active compounds are ideal for IVD regeneration. It is important to note that PRP does, according to various studies [15, 16], both in vitro and in vivo, offer a milieu for tissue repair and wound healing. Leukocytes are one of the essential components of PRP, but despite their widespread usage in regeneration, there is no agreed-upon procedure for its manufacture [17, 18]. It has been demonstrated that PRP with large concentrations of leukocytes can cause an inflammatory response and extracellular matrix breakdown due to the production of pro-inflammatory cytokines, including IL-1β and TNF-α, eventually activating the NF-κB signaling pathway [19, 20]. Pure platelet-rich plasma (P-PRP) is created to minimize the adverse effects of leukocytes and gain the most significant therapeutic benefits in light of the unfavorable effects of leukocyte platelet-rich plasma (L-PRP) on tissue healing [21]. Due to its exclusion of leukocyte intervention, which has been confirmed [20], P-PRP would be preferable for the early IVDD stage. NPCs play a crucial role in IVD and are responsible for self-renewal and self-regeneration, particularly when stimulated by PRP. Studies have shown that PRP has superior effects on reducing the progression of IVDD, even though the processes are not yet fully understood [22].

According to research, the secreted protein known as semaphorin-3A (Sema3A) is extensively expressed in a healthy disk and significantly reduced during degeneration [23]. Sema3A also contributes significantly to maintaining IVD homeostasis due to its ability to prevent angiogenesis and abnormal innervation [24, 25]. However, further investigation is required to better understand how platelet-rich plasma (PRP) can regulate Sema3A and its involvement in the development of IVDD. In order to compare the effects of P-PRP and L-PRP on NP cells, as well as to investigate the interactions between Sema3A, PRP, and the NF-κB signaling pathway in the process by which PRP attenuates IVDD in well-established IL-1β-induced IVD degenerative cell models and organ models, the current study’s goal is to provide novel insight for IVDD treatment.

## Materials and methods

### Preparation of pure platelet-rich plasma and leukocyte platelet-rich plasma

**Fig. 1:**
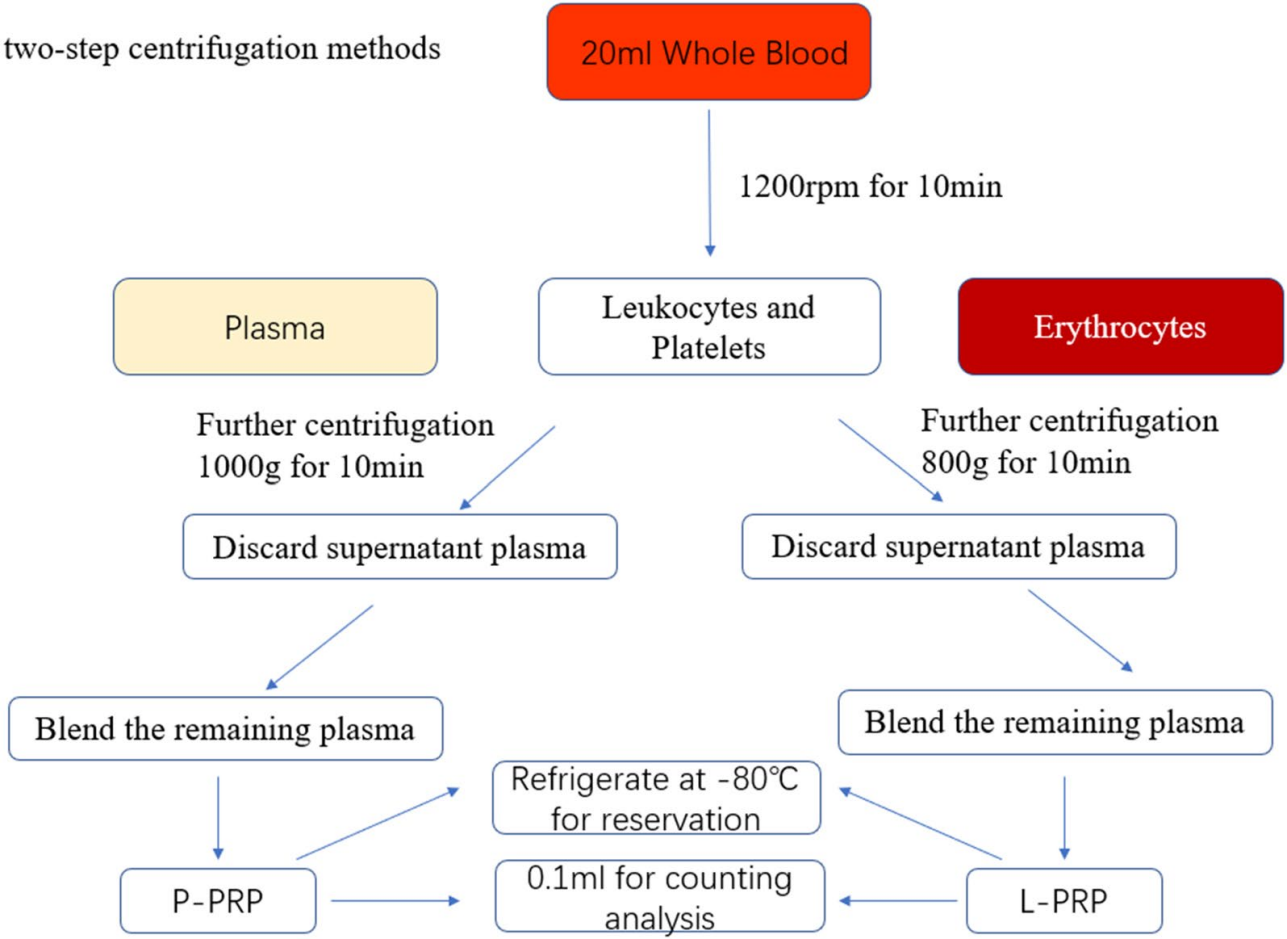
Flowchart for the two-step centrifugation process used to create pure platelet-rich plasma (P-PRP) and leukocyte platelet-rich plasma (L-PRP)

The Institutional Animal Care and Use Committee, LTD Laboratory Animal Ethics Committee of Kangtai Medical Laboratory Services Hebei Co., approved the study (approval number: MDL2022-11-07-01). The two-step centrifugation technique created P-PRP and L-PRP (Fig. 1). A sodium heparin anticoagulation tube was used to collect the whole blood, which was then centrifuged at 1200 rpm for 10 min to separate it into three fractions: erythrocytes at the bottom, leukocytes and platelets at the junction, and plasma at the top. The upper layer, which contained the plasma and platelet fractions, was carefully aspirated, after which subsequent centrifugation at 1000 g for 10 min took place. The remaining plasma was combined with precipitated platelets and thoroughly blended to create P-PRP after supernatant plasma (platelet-poor plasma) was discarded. L-PRP was made using techniques identical to those used to make P-PRP. After being centrifuged at 1200 rpm for 10 min, the fresh whole blood was briefly divided into the three layers mentioned above. The top two layers were then centrifuged at 800 g for 10 min. After a second centrifugation, the supernatant plasma (platelet-poor plasma) was removed, and the leftover plasma was added to the deposited platelets and thoroughly mixed to obtain L-PRP. Both P-PRP and L-PRP were treated with lyophilized thrombin and 10% calcium chloride in an appropriate ratio, and both types of PRP were then left at room temperature for 1 h to activate. The activated products were then extracted by spinning at 2000 rpm for 10 min and stored at − 80 °C until needed.

### Component analysis of whole blood, P-PRP and L-PRP

Using an automatic hematology analyzer, platelets and leukocytes in P-PRP, L-PRP, and whole blood were counted and analyzed. Following the guidelines provided by the respective manufacturer, an ELISA kit was used to measure the levels of TNF-α (ygyr biotech, MM0180R2, China), IL-1β (mibio, ml003057-2, China), TGF-β (mibio, ml002856-2, China), PDGF (mibio, ml00314-2, China), and VEGF (mibio, ml002862-2, China).

### NPCs isolation and culture

Beijing Huafukang Biotechnology Co., Ltd., provided 8-week-old male Sprague-Dawley (SD) rats for the primary rat NPCs (Approval Number. SCXK2019-0008). After anesthesia, the rats were killed and set down on a clean surface. Following standard cleaning and disinfection, the entire lumbar vertebra was removed. The IVD was then exposed, and the soft tissues and cartilage endplates were peeled off to reveal the NP tissue. This tissue was then cut into 0.5 mm^3^ pieces with ophthalmic scissors and digested with 0.2% type II collagenase (meilunbio, MB2665, Dalian, China) at 37 °C for 4 h to harvest NPCs. Then, NPCs were incubated at 37 °C and 5% CO_2_ in DMEM/F12 media (Gibco, Thermo Fisher Scientific, Inc., USA) with 20% fetal bovine serum. Every 3 days, the cultural media was replaced. For the subsequent studies, cells from the second generation were obtained.

### PRP injection into IVDs and organ culture

Rats were used to extract the whole vertebral body-disk-vertebral body segments of the Co6/7 and Co7/8, along with the upper and lower cartilage endplates fused with IVD. The IL-1β was taken for inducing the IVDD organ model after the units were put into a 6-well plate. Then, while being watched, a 21G needle was used to inject 20ul of PRP into the experimental groups while receiving the same volume of PBS into the control groups. The two groups were compared at 7, 10, and 14 days after the injections. Finally, HE and IHC methods were used to examine the morphologic results.

### Histological staining

All IVDs were removed from the vertebral body-disk-vertebral body unit and preserved for 24 h in 4% paraformaldehyde before being decalcified with EDTA. The tissues were dehydrated using graded ethanol before being embedded in paraffin and divided into slices of 5 mm thickness for staining. After deparaffinization and rehydration, the sections were subjected to hematoxylin and eosin staining. Under a light microscope, the process of morphologic observation began.

### Immunohistochemical staining

To deactivate endogenous peroxidases, the paraffin slices were treated with 3% H_2_O_2_ at room temperature for 10 min prior to deparaffinization and dehydration. The slices were placed in a sodium citrate buffer solution to repair the antigen and cooked for 15 min. The primary antibody was then administered, followed by blocking with 5% bovine serum for 15 min in a refrigerator set at 4 °C overnight. The samples were cleaned with PBS before being treated with secondary antibodies for 30 min at room temperature. Under the microscope, positive staining results were observed using a freshly prepared DAB solution, while counterstaining with hematoxylin staining solution allowed for visualization of contrasting cellular structures. The specimens were dehydrated using 100% and 95% ethanol before being immersed in xylene for one minute and sealed with neutral resin. The light microscope was ultimately used for imaging and analysis.

### Immunofluorescence staining

After being cultivated to 90% cellular fusion, NPCs were implanted into a 24-well plate. The sample was cleaned with PBS, fixed with 4% paraformaldehyde for 15 min at room temperature, and then cleaned three times with PBS. The samples were then permeabilized for 15 min at room temperature using 0.5% Triton-X 100. The primary antibodies were diluted in TBST according to the manufacturer’s recommendations before being introduced to the specimens for overnight incubation at 4 °C after the cells had been PBS-washed. A fluorescent secondary antibody was chosen to react with the cells for 2 h at 37 °C after the cells had been washed. The DAPI was added and left to sit for 5 min at room temperature. The anti-fluorescent quencher was eventually added dropwise before the glass slides were put on top of the slides. A fluorescence microscope was used to see the fluorescence images.

### EDU assay

NPCs were seeded into a 96-well plate, and EDU culture media was added for 2 h afterward. After that, samples were fixed using 4% paraformaldehyde in PBS for 30 min at room temperature. Following a PBS wash, the Apollo staining solution was added dropwise, and the samples were then incubated for 30 min at 37 °C in a photophobic environment. The slides were then treated with Hoechst33342 solution and incubated for 30 min at 37 °C in a dark box. After washing, a laser scanning confocal microscope was used to capture cell proliferation images, which were then quantitatively analyzed using the program image J.

### TUNEL assay

Using the ApopTag InSitu apoptosis detection kit (biyuntian, C1090, China), the Terminal deoxynucleotidyl Transferase-Mediated Nick End Labeling (TUNEL) test was carried out to determine the apoptosis NPCs following the manufacturer’s protocol. In a nutshell, paraffin sections underwent multiple processes of deparaffinization, hydration, and antigen retrieval before being permeabilized for 20 min at room temperature using DNase-free Proteinase K. Afterward, TUNEL reaction solution was added to the samples, which were then incubated for 1 h at 37 °C in a dark, moist atmosphere. Cell nuclei were then counterstained while DAPI was present. After applying an anti-fluorescent quencher, the sections were examined under a confocal microscope.

### Western blot analysis

NPCs were lysed with RIPA buffer containing protease and phosphatase inhibitors to extract total proteins. Bicinchoninic acid (BCA; MDL, MD913053, Hebei, China) working solution was prepared to determine protein concentration. Total protein was electrophoresed in a sodium dodecyl sulfate polyacrylamide gel electrophoresis (SDS-PAGE; Bio-Rad, MA, USA) and then transferred to a polyvinylidene fluoride (PVDF; Millipore, Billerica, MA, USA) membrane. The membrane was blocked with 5% skimmed milk in TBST (Beyotime) for 2 h at room temperature and reacted with primary antibody against collagen II (1:3000; AF0135; Affinity), aggrecan (1:3000; DF7561; Affinity), MMP-3 (1:2000; 340612; ZEN BIO), ADAMTS-4 (1:2000; bs-4191R; Bioss), p65 (1:1000; R25150; ZEN BIO), p-p65 (1:1000; 310013; ZEN BIO), CD31 (1:1000; 28083-1-AP; Proteintech), NF200 (1:1000; 18934-1-AP; Proteintech), Sema3A (1:1000; DF8609; Affinity), and β-actin (1:1000; #AF7018; Affinity) at 4 °C overnight. After washing by TBST, the membrane was incubated with HRP goat anti-rabbit IgG (1:1000; 511103; ZEN BIO) for 1 h at room temperature. The protein was detected with an enhanced chemiluminescence (ECL; UVP, GelDoc-It310, MA, USA) reagent, and the chemiluminescence imaging system (Bio-Rad, 170-8280, MA, USA) was taken for imaging.

### Quantitative real-time fluorescence polymerase chain reaction

TRIzol reagent was used to extract the total RNA from NPCs, and cDNA was produced by reverse transcription afterward. For mRNA analysis, SYBR Green was used in quantitative real-time fluorescence PCR (RT-qPCR). Using the 2^−ΔΔCT^ formula, the relative expression of each target mRNA was calculated. The top target gene sequences used in the current investigation are listed in Table 1. The expression of β-actin was used as a benchmark for quantitative analysis.

**Table 1:** Primers used in quantitative real-time polymerase chain reaction for gene expression analysis

Gene	Primer sequence (5′–3′)
Collagen II-F	CGCCATGAAAGTCTTCTGCAACA
Collagen II-R	CACCAGTTCTTCCGAGGCACA
Aggrecan-F	CGCTGGTCTGATGGACACTC
Aggrecan-R	AGATCATCACTACGCAGTCCT
ADAMTS-4-F	GCTGTGATCGAATCATTGGCT
ADAMTS-4-R	GACCACATCGCTGTATCCGT
MMP-3-F	TGTCTTTGAAGCATTTGGGTTT
MMP-3-R	GTTGCTCTTCAATATGTGGGT
Sema3A-F	AGGACTCACATTTTGAAAACGG
Sema3A-R	GATAGCAAAGTCTCGTCCCAT
CD31-F	AGGTGCTATTCTATAAGGACGAT
CD31-R	TGTTCAGTATCACGGTGCATT
NF200-F	AGGACCGTCATCAGGTAGACA
NF200-R	TCCAGGGCCATCTTGACGTTG
P65-F	GGGTACATCCGATCCATACGTC
P65-R	ACCTAATTCCGAGTAGGGCAC
β-actin-F	CTGAACGTGAAATTGTCCGAGA
β-actin-R	TTGCCAATGGTGATGACCTG

### Transmission electron microscopy (TEM)

NPCs were collected for centrifugation and then fixed for 2 h in 2.5% glutaraldehyde. The samples were fixed in 1% osmic acid at 4 °C for 2 h after being washed three times with phosphate buffer. The NPCs were then put into a model for polymerization after being dehydrated with graded ethanol, implanted in Epon-Araldite resin for penetration, and so on. The ultrathin sections were created using an ultramicrotome, and they were counterstained with 2.7% lead citrate and 3% uranyl acetate. In the end, Hitachi HT7800 transmission electron microscopy was used to view the microstructures of the sections.

### Statistical analysis

Statistical data were analyzed using SPSS 22.0 software (IBM Corp., Armonk, NY, USA), and graphs were generated using GraphPad Prism version 9.0 (GraphPad Software Inc., La Jolla, CA). All results were regarded as mean ± standard deviation (SD) with at least three replicative experiments. Student’s t test was used to compare two groups, and one-way analysis of variance (one-way ANOVA) was applied for multiple comparisons. The *p* value less than 0.05 was considered as a statistical significance.

## Results

### Characterization of L-PRP and P-PRP

**Fig. 2:**
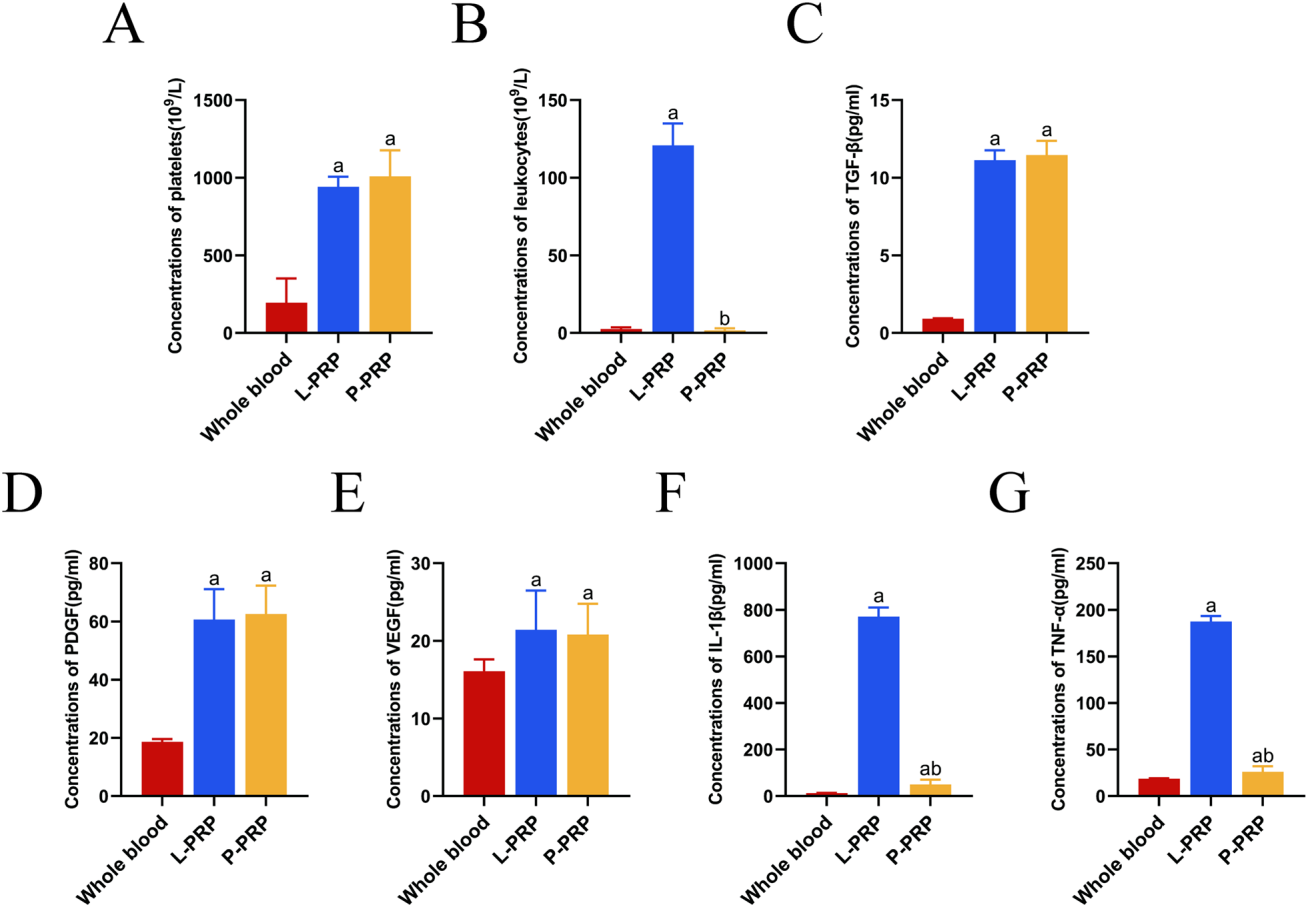
Composition of whole blood, L-PRP, and P-PRP. **A**, **B** Concentrations of platelets and leukocytes. **C**–**G** Concentrations of TGF-β, PDGF, VEGF, IL-1β, and TNF-α. ^a^*p* < 0.05 versus whole blood; ^b^*p* < 0.05 versus L-PRP

Both L-PRP (942 × 10^9^/L) and P-PRP (1008.5 × 10^9^/L) exhibited comparable platelet concentrations, which were nearly five times greater than those observed in whole blood (195 × 10^9^/L) (Fig. 2A). However, the leukocyte concentrations in the three groups showed substantial differences, while the leukocyte concentration in the entire blood was 2.625 × 10^9^/L, compared to 120.833 × 10^9^/L in L-PRP and 1.775 × 10^9^/L in P-PRP (Fig. 2B). TGF-β, PDGF, and VEGF levels were also comparable across the two types of PRP and were higher than those found in whole blood (Fig. 2C–E). Interestingly, whole blood had significantly lower TNF-α and IL-1β levels than PRP, especially P-PRP (Fig. 2F, G). These findings showed that P-PRP is preferable to L-PRP because it produces less pro-inflammatory cytokines due to its lower leukocyte content and similar ability to enrich platelets and growth factors.

### Isolation and culture of NPCs and IVD tissues

**Fig. 3:**
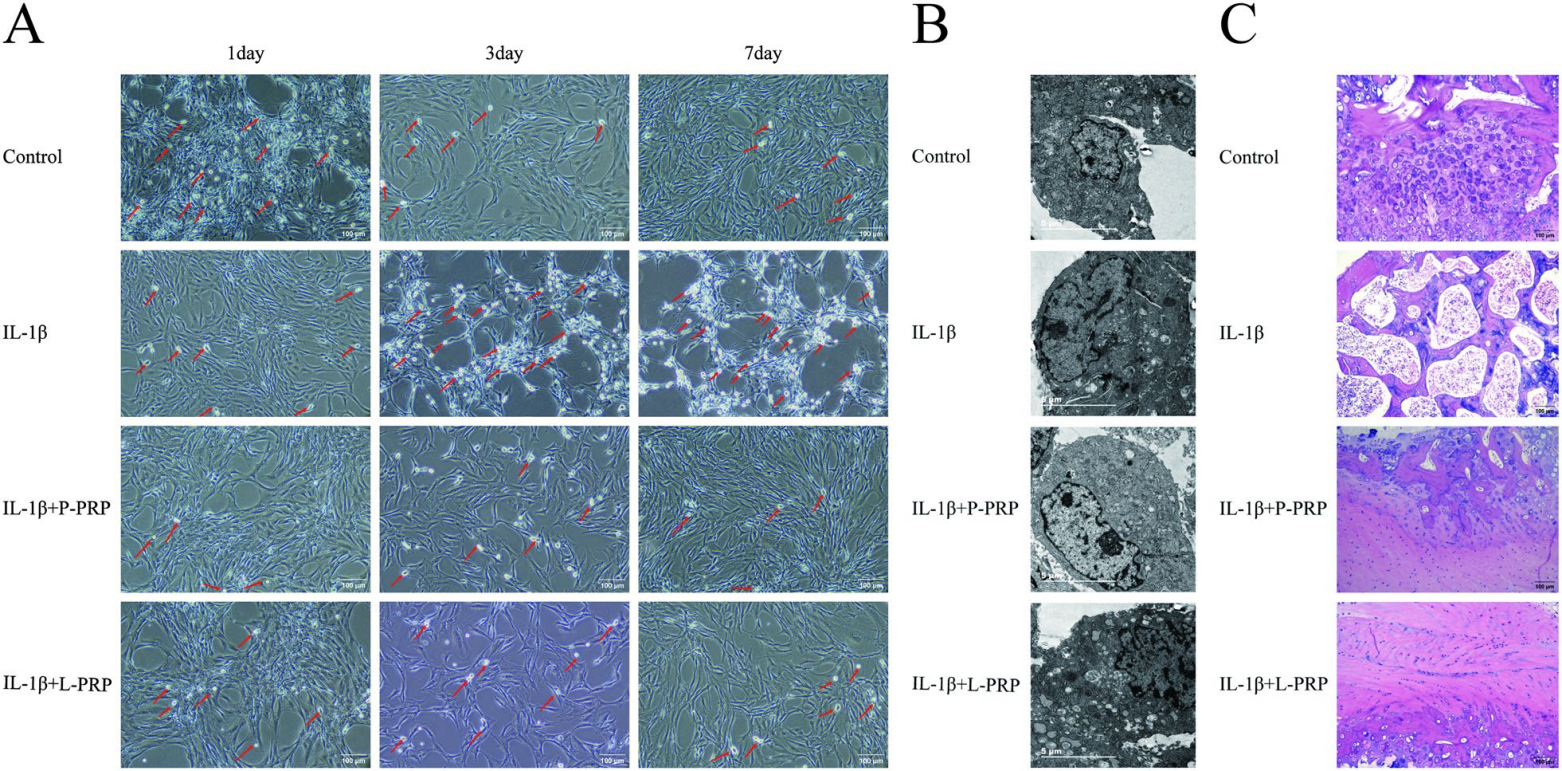
The effect of PRP on NPCs culture. **A** Both types of PRP enhanced the number of NPCs induced by IL-1β (Bar = 100 μm), and the dead cells were pointed by red arrows. **B** The microstructures of NPCs were observed under a Transmission electron microscopy (Bar = 5 μm). **C** HE staining was used to detect the pathological changes of IVD tissues (Bar = 100 μm)

The NPCs were isolated from the disks after the NP tissues were taken from healthy rat IVD. It has been claimed that 10 ng/ml IL-1β in vitro may consistently create IVDD models [26]. As a result, 10 ng/ml IL-1β was chosen for the following trials. NPCs were treated with PBS as a control. Treatment with IL-1β resulted in a noticeable change in cell morphology characterized by altered cell shape, as well as a significant reduction in the number of NPCs, which were observed under light microscopy (Fig. 3A). In addition, transmission electron microscopy was used to investigate the microstructures of NPCs that IL-1β generated. According to Fig. 3B, increased autophagic vacuoles are seen in rat NPCs when IL-1β is present, which substantially impacts the pathological development of IVDD. Notably, Fig. 3C demonstrates that PRP supported the healing of IVD tissues and preserved the fundamental features of IVD tissues harmed by IL-1β. These findings suggested that PRP reduced IVDD brought on by IL-1β and encouraged autophagy in NPCs, which may slow the progression of degenerative disease.

### Effects of PRP on cell proliferation and apoptosis

**Fig. 4:**
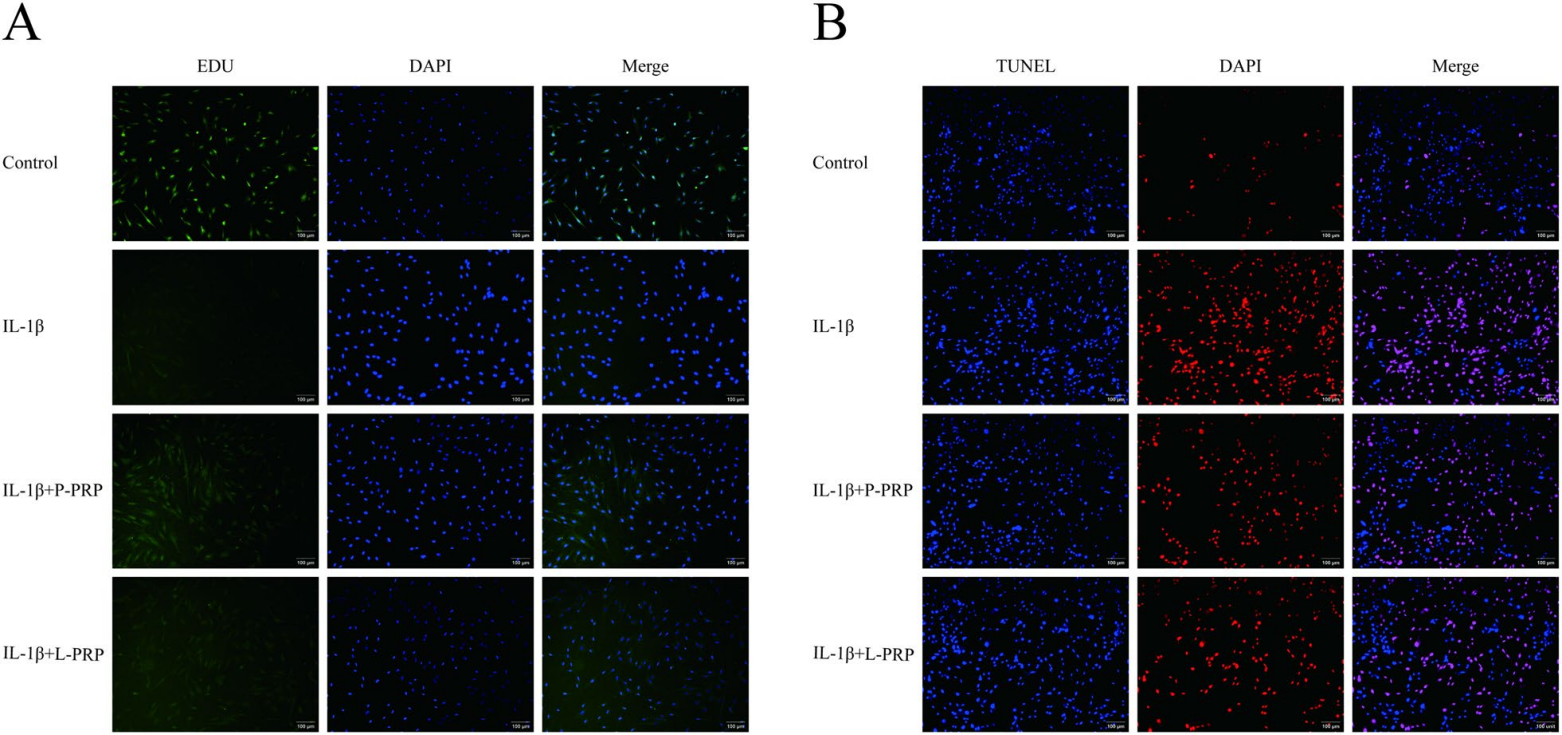
PRP enhanced the proliferation of NPCs induced by IL-1β and mitigated NPCs apoptosis (Bar = 100 μm). **A** The proliferation of NPCs was detected by EDU assay. **B** The apoptosis of NPCs was observed by TUNEL assay (Bar = 100 μm)

Before being stimulated with IL-1β, NPCs were first treated for 24 h with L-PRP and P-PRP, respectively. Additionally, an EDU experiment (Fig. 4A) was carried out to investigate the proliferative potential of NPCs following L-PRP and P-PRP therapy. Figure 4 demonstrates that P-PRP was more advantageous for NPC growth than L-PRP. The TUNEL test (Fig. 4B) was created to further show that IL-1β treatment enhanced apoptosis in NPCs, whereas PRP reduced the amount of IL-1β-induced apoptotic cells. In contrast to L-PRP, P-PRP demonstrated a pronounced ability to mitigate IL-1β-induced apoptosis and significantly enhance proliferation and cell survival in NPCs.

### PRP suppressed IL-1β-induced matrix degradation in rat NPCs

**Fig. 5:**
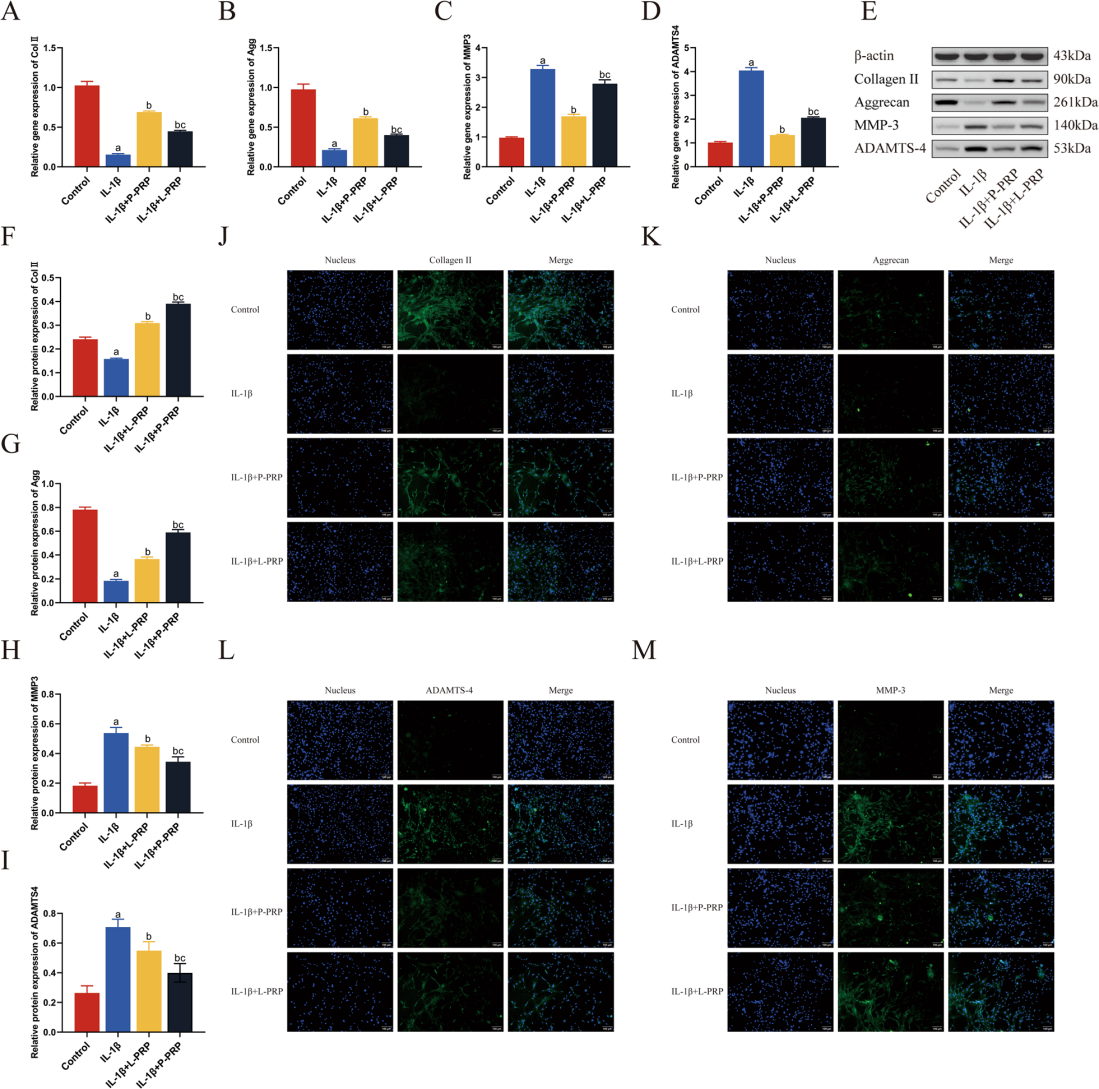
PRP-ameliorated ECM degradation in IL-1β-induced NPCs. **A**–**D** The gene expression of collagen II, aggrecan, MMP-3, and ADAMTS-4 in NPCs was analyzed by Quantitative real-time PCR. **E**–**I** The western blot assays were used to detect the protein expression. **J**–**M** Immunofluorescence staining was further taken for positive expression (Bar = 100 μm). ^a^*p* < 0.05 versus control; ^b^*p* < 0.05 versus NPCs treated with IL-1β only; and ^c^*p* < 0.05 versus NPCs treated with IL-1β + P-PRP

Since PRP increases proliferation while decreasing apoptosis in IL-1β-induced NPCs, PRP might decrease cell apoptosis by slowing the breakdown of extracellular matrix components. According to RT-qPCR data, IL-1β upregulated the expression of matrix catabolic enzymes such as MMP-3 and ADAMTS-4 while downregulating the production of aggrecan and collagen II (Fig. 5C, D). However, aggrecan and collagen II expression were significantly increased following PRP modulation, while the expression of matrix catabolic enzymes was significantly decreased (Fig. 5A, B). Additionally, a western blot study showed that PRP boosted the expression of the proteins aggrecan and collagen II while inhibiting the production of the proteins for the matrix catabolic enzymes (Fig. 5E–I). Immunofluorescence labeling revealed that IL-1β decreased matrix synthesis, whereas PRP reduced IL-1β-induced matrix breakdown (Fig. 5J–M). In other words, these findings showed that P-PRP exerted a more substantial influence than L-PRP on reducing matrix deterioration.

### PRP attenuated IL-1β-induced Sema3A activity inhibition in rat IVD tissue

**Fig. 6:**
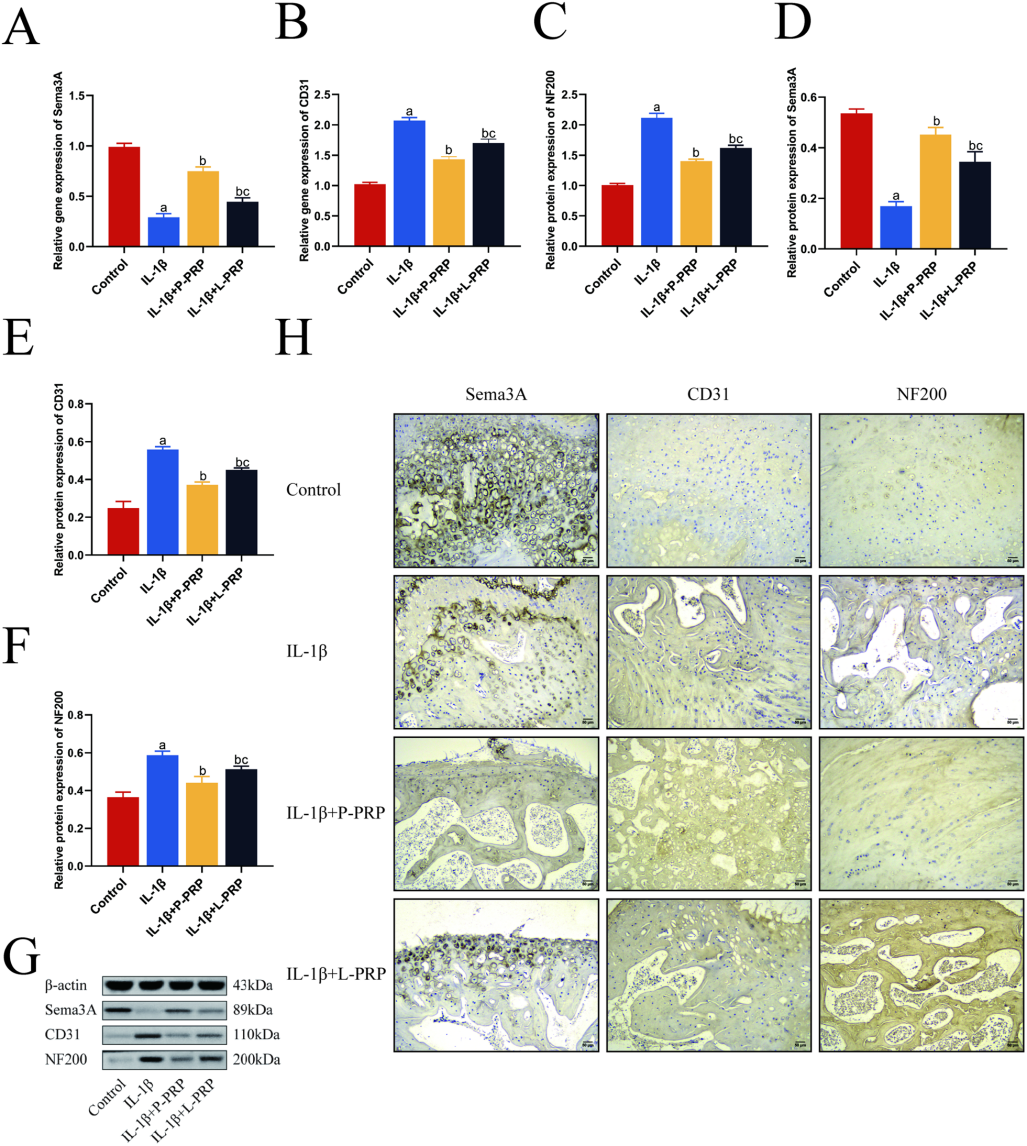
PRP improved Sema3A activity in rat IVD tissue induced by IL-1β. **A**–**C** RT-qPCR was used to detect the effect of L-PRP and P-PRP on the mRNA expression of Sema3A, CD31, and NF200. **D**–**G** The protein expression was observed by western blot assay (Bar = 100 μm). **H** The positive expression was tested by immunohistochemical staining (bar). ^a^*p* < 0.05 versus control; ^b^*p* < 0.05 versus NPCs treated with IL-1β only; ^c^*p* < 0.05 versus NPCs treated with IL-1β + P-PRP

Sema3A expression was downregulated during the IVDD process, and IL-1β was crucial in stifling Sema3A activity. Before the IL-1β-induced IVDD, L-PRP and P-PRP were introduced to study the influence of Sema3A. As previously demonstrated, IL-1β stimulation increased the expression of MMP-3 and ADAMTS-4 genes while inhibiting Sema3A gene expression. PRP, however, reversed this effect and decreased the expression of the ECM as mentioned above degradation markers, demonstrating that PRP significantly reduced the effect of IL-1β. RT-qPCR was used to find Sema3A, CD31, and NF200 mRNA expression (Fig. 6A–C). Since innervation and angiogenesis are related to sema3A activity [27], NF200 and CD31 were also identified using western blot and immunohistochemistry labeling. The results of a western blot examination (Fig. 6D–G) and subsequent densitometric analyses showed that the expression of the proteins NF200 and CD31 significantly increased when IL-1β was present. Nevertheless, it should be noted that P-PRP led to a reduction in the protein expression of NF200 and CD31 that was induced by IL-1β. Additionally, immunohistochemical labeling showed that PRP reversed IL-1β-induced NF200 and CD31 protein overexpression (Fig. 6H). These findings revealed that PRP, particularly P-PRP, effectively restored IL-1β-induced reduction of Sema3A activity during IVDD.

### PRP attenuated the activity of NF-κB signaling pathway and promoted Sema3A expression in rat NPCs

**Fig. 7:**
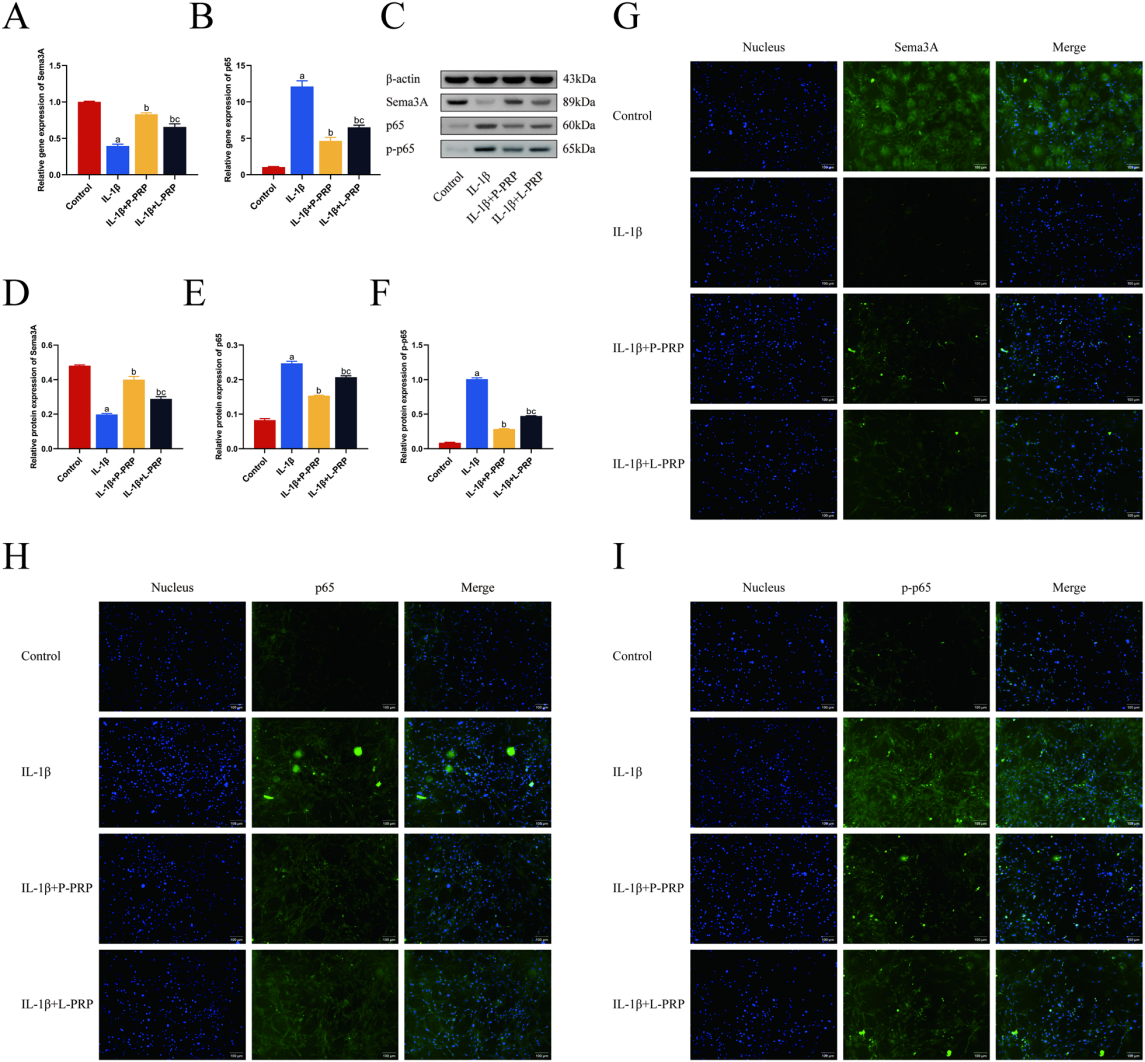
Inhibition of NF-κB pathway exerted a protected effect on IL-1β-induced NPCs and promoted Sema3A activity. **A**, **B** Real-time PCR showed that both L-PRP and P-PRP increased the mRNA of Sema3A and dwindled p65 expression. **C**–**F** Western blotting analyzed that the increase in p65 phosphorylation/p65 ratio induced by IL-1β was inhibited by PRP. **G**–**I** Immunofluorescence staining showed that P-PRP distinctly suppressed NF-κB pathway and increased Sema3A expression (Bar = 100 μm). ^a^*p* < 0.05 versus control; ^b^*p* < 0.05 versus NPCs treated with IL-1β only; and ^c^*p* < 0.05 versus NPCs treated with IL-1β + P-PRP

Given the importance of NF-κB signaling pathway in IVDD and inflammation [28], the effect of NF-κB signaling pathway during the regulation of L-PRP and P-PRP on Sema3A was assessed. The expression of p65, a key protein associated with NF-κB signaling pathway, was assessed using both RT-qPCR and western blot techniques following treatment with both L-PRP and P-PRP. Figure 7A, B illustrates how RT-qPCR evidence demonstrates that IL-1β enhanced the expression of the p65 gene, whereas PRP prevented this effect. Furthermore, p65 translocation in the nucleus of NP cells increased in the presence of IL-1β, and PRP reduced the IL-1β-induced p65 translocation, limiting the impact of IL-1β (Fig. 7C–F). In addition, immunofluorescence labeling of phosphorylated p65 (Fig. 7G–I) was used to show that PRP reversed IL-1β-induced p65 translocation and increased p65 phosphorylation. Our findings showed that P-PRP had a more pronounced effect, whereas both kinds of PRP raised Sema3A expression and ultimately inhibited NF-κB signaling pathway.

## Discussion

PRP therapy has shown favorable responses in various disorders, such as osteoarthritis, IVDD, and osteogenesis [12, 15, 29]. In recent years, PRP has received a great deal of attention in both basic science and clinical studies because of its impact on tissue and organ regeneration and repair [16]. Currently, numerous studies have shown that PRP is a better biological tissue regeneration and repair treatment [30–33]. Both L-PRP and P-PRP were influential in the current investigation of reducing ECM degradation and relieving Sema3A activity inhibition brought on by IL-1β, which was crucial in preventing the development of IVDD in rats. Additionally, PRP had a protective effect against IVDD via increasing Sema3A activity by inhibiting NF-κB signaling pathway. However, varied PRP preparation methods led to variable PRP components. Due to the pro-inflammatory action of PRP, leukocytes, for instance, have been proposed to be eliminated in musculoskeletal mending applications [17, 19, 34]. To explore the underlying mechanism and evaluate the potential superiority of P-PRP as a treatment option for IVDD, this study focused on generating P-PRP and comparing it with L-PRP. The production of L-PRP and P-PRP in this study was done using a two-step centrifugation procedure. L-PRP and P-PRP were both abundant in the platelet. However, due to a lower concentration of leukocytes and inflammatory stimuli, P-PRP significantly impacted the proliferation of NP cells.

The preservation of the anabolic and catabolic balance of the ECM in NP tissue ensures the maintenance of osmotic pressure and IVD elasticity [35]. IVDD may be brought on by the breakdown of aggrecan and collagen II when an inflammatory response occurs [36, 37]. Pro-inflammatory agents like IL-1β and TNF-α are strongly linked to catabolism because they boost the production of matrix catabolic enzymes, including MMPs and ADAMTSs [38, 39]. In a rat model of herniated lumbar disks, IL-1β was administered directly into the ruptured disks, after which degeneration and inflammation were noted [26]. PRP was created to reduce inflammation and encourage the production of ECM components to slow the progression of IVDD; in addition to the growth factors or cytokines listed above, additional components such as PDGF, TGF-β, and IGF-1 also exist within PRP [14, 40]. PRP, an autologous serum extract rich in platelets and growth factors, is frequently used to treat inflammatory illnesses such as osteoarthritis and synovitis due to its anti-inflammatory properties [41, 42]. Notably, in the initial stages of IVDD, L-PRP and P-PRP have been shown to decrease MMP-1 and MMP-13 while boosting aggrecan and collage II [20]. PRP, particularly P-PRP, simultaneously encouraged NPMSCs development and extracellular matrix-related protein synthesis [21]. In this regard, our findings demonstrated that PRP effectively mitigated the IL-1β-induced matrix breakdown and promoted the growth of NPCs.

A healthy IVD is avascular; however, when IVD is vulnerable to degeneration, blood vessels and nerve fibers may extend pathologically from the outer region into the inner region of the NP, which causes low back pain and manifests as a high-intensity zone on MRI [43, 44]. Initially identified as an inhibitor of neurite outgrowth in the brain system and as a barrier to endothelial cell survival and vascular expansion, Sema3A is a secreted protein [45, 46]. According to studies, Sema3A lowered the dorsal root ganglion’s axon development and decreased expression in deteriorated disks [47]. By connecting with NRP (Neuropilin-1) and Plexin, Sema3A achieved its biological effects, such as cell migration, angiogenesis, immunological modulation, and bone metabolism [48]. Additionally, several researchers hypothesized that exogenous Sema3A application could diminish NGF expression in the deteriorated NP tissues, limiting nerve growth into the degenerated disks and significantly improving chronic lower back pain [23, 24, 27]. Furthermore, after IL-1β treatment in NPCs, the upregulation of inflammatory proteins in deteriorated disks decreased the expression of Sema3A.

Moreover, earlier research has shown that many signaling pathways, including NF-κB signaling pathway, c-Jun/JNK signaling pathway, MAPK signaling pathway, and others, were involved in the development of IVDD. The cytoplasm of eukaryotes frequently contains the nuclear transcription factor known as NF-κB [28, 49, 50]. One of the most prevalent forms in mammals, the P65 dimer complex, is crucial for regulating apoptosis, proliferation, and inflammatory response [28]. Previous research has demonstrated that several inflammatory-related disorders can be linked to the activation of the NF-κB signaling pathway [51]. In contrast, NF-κB is inactive in the cytoplasm of resting cells. NF-κB was quickly transported into the nucleus after being induced by numerous inflammatory agents, such as IL-1β, IL-6, and TNF-α, and it then controlled the transcription and expression of target genes [37, 39]. Numerous studies have demonstrated a favorable correlation between the degree of IVDD and the expression levels of IL-1β and NF-κB in deteriorated disk tissue [28, 52]. Furthermore, our earlier findings showed that IL-1-induced NF-κB signaling pathway activation decreased Sema3A expression in NPCs. The current study also showed that L-PRP and P-PRP, particularly P-PRP, significantly reduced the activation of the NF-κB signaling pathway brought on by the treatment with IL-1β. This was determined by looking for p65 and p65 phosphorylated in the proteins using western blotting and immunofluorescence staining.

PRP therapy is a very effective therapy in many aspects. The use of P-PRP and L-PRP in the treatment of IVDD and their probable mechanisms were thoroughly examined at the cellular level in this study. However, the well-established cell degeneration models have limitations that prevent them from accurately simulating the complete process; hence, future relevant animal investigations should be conducted. Furthermore, rat NP cells were employed for analysis in this work, which may deviate somewhat from the actual clinical effect. The specific mechanism should also be thoroughly studied, particularly the interaction between the IL-1β, Sema3A, and NF-κB pathways.

**Fig. 8:**
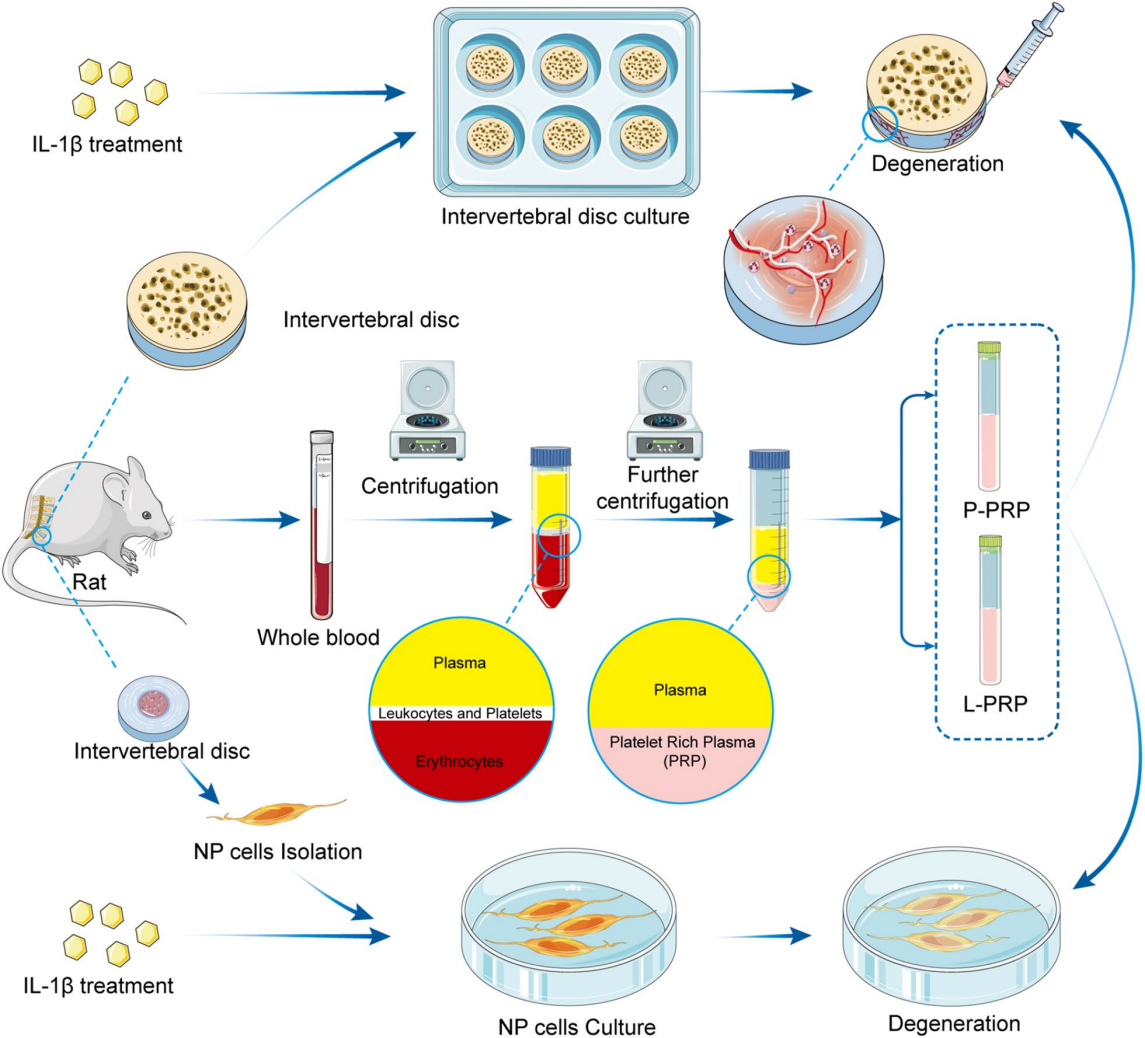
Graph in a schematic. The isolation and culture of rat NPCs and IVDs. The next step was to introduce IL-1β to cause degeneration. L-PRP and P-PRP, later employed to treat IL-1β-induced degenerative IVDs and NPCs, were synthesized using two-step centrifugation techniques

This study showed that both L-PRP and P-PRP stimulated the growth of NPCs cultivated in vitro. However, because leukocytes were absent, P-PRP was more effective at reducing ECM breakdown and inflammatory response. The NF-κB signaling system was noticeably stimulated by concentrated leukocytes in L-PRP, which set off an inflammatory cascade. Based on our research, we concluded that P-PRP significantly reduced IVDD through increasing Sema3A expression. Additionally, those results and our earlier discoveries showed a connection between the IL-1β, Sema3A, and NF-κB signaling pathways in rat NPCs. In any case, these findings provide insights into a potential mechanism through which P-PRP exerts its protective effects against IVDD. These results support the exploration of a novel approach for the prevention and treatment of IVDD (Fig. 8).

## Abbreviations

PRP: Platelet-rich plasma
IVDD: Intervertebral disk degeneration
P-PRP: Pure platelet-rich plasma
Sema3A: Semaphorin-3A
NPCs: Nucleus pulposus cells
L-PRP: Leukocyte platelet-rich plasma
ECM: Extracellular matrix
DDD: Degenerative disk diseases
NP: Nucleus pulposus
AF: Annulus fibrosus
IVD: Intervertebral disk
PDGF: Platelet-derived growth factor
TGF-β: Transforming growth factor-β
VEGF: Vascular endothelial growth factor
IGF-1: Insulin-like growth factor-1
bFGF: Basic fibroblast growth factor
MMP: Matrix metalloproteinases
ADAMTS: A disintegrin and metalloproteinase with thrombospondin motif
